# Effects of a Structured Parent Training Program on Parents’ Active Mediation of Adolescents’ Smartphone Use: A Controlled Experimental Study

**DOI:** 10.3390/bs16030452

**Published:** 2026-03-19

**Authors:** Yan Chen, Qiongying Wu, Canyu Hu, Qian Gu, Hongshan Gu, Chuanhua Gu, Yuqi Cao

**Affiliations:** 1 Tin Ka Ping College of Education, China Three Gorges University, Yichang 443002, China; chenyan@ctgu.edu.cn (Y.C.); wuqiongying.12@gmail.com (Q.W.); 13886169194@163.com (C.H.); 2 Key Laboratory of Adolescent Cyberpsychology and Behavior (CCNU), Ministry of Education, Wuhan 430079, China; 3 Key Laboratory of Human Development and Mental Health of Hubei Province, School of Psychology, Central China Normal University, Wuhan 430079, China; 4 School of Educational Science, Liaocheng University, Liaocheng 252059, China; guqian2023@163.com; 5 College of Liberal Arts and Sciences, DePauw University, Greencastle, IN 46135-0037, USA; hongshangu_2029@depauw.edu; 6 School of Humanities, Political Science and Law, Henan University of Engineering, Zhengzhou 451191, China

**Keywords:** parental active mediation, parent training, experimental study, adolescent, problematic smartphone use

## Abstract

Problematic smartphone use among adolescents can lead to various negative consequences. Parental active mediation has been identified as an effective strategy to mitigate these problems. In response, numerous parent training programs have been developed. However, it remains unclear whether these programs can effectively enhance parents’ active mediation. A 2 (group: experimental vs. control) × 3 (time: pretest, post-test, follow-up) mixed factorial design was employed to examine the effects of parent training on parents’ active mediation. A total of 60 parents of adolescents with problematic smartphone use participated in the experiment. Parents in the experimental group attended a six-session parent training program, while those in the control group received no intervention. Parental active mediation was assessed using a validated self-report scale at all three time points. Results indicated significant main effects of group and time, as well as a significant group × time interaction. Among parents with the same initial level of active mediation, those who participated in the training demonstrated higher levels of active mediation than those in the control group, and this improvement was maintained two months later. These findings provide empirical evidence that parent training can sustainably enhance parents’ active mediation in managing adolescents’ smartphone use.

## 1. Introduction

With the rapid development of mobile digital media, smartphones have become the dominant tool through which adolescents engage in entertainment, acquire information, and exchange opinions. Data from the Sixth National Survey on Internet Use by Minors indicate that 97.3% of Chinese minors access the internet primarily via smartphones. While moderate smartphone use can positively contribute to adolescents’ learning, social engagement, and well-being (Abbasi et al., 2021), excessive or poorly regulated use has been linked to problematic smartphone use—characterized by cognitive and behavioral symptoms of addiction such as preoccupation, loss of control, withdrawal, and functional impairment (Elhai et al., 2017; Van Deursen et al., 2015). This pattern of use is associated with increased anxiety, depressive symptoms, sleep disturbances, and academic difficulties (Elhai et al., 2017; Fabio et al., 2022; Kates et al., 2018). Given these risks, scholars have increasingly emphasized the importance of family-level protective factors, particularly parental mediation, in shaping adolescents’ digital media experiences.

In the modern era of new media, researchers have outlined three key types of parental mediation: (a) active mediation, in which parents offer guidance on media content and usage through interactive forms such as discussions and explanations; (b) restrictive mediation, where parents establish limits on their children’s media use; and (c) parental supervision, where parents keep a close watch on their children’s media consumption, including checking emails and websites (Nikken & Jansz, 2014). This framework has been elaborated in subsequent theoretical models emphasizing that active mediation supports autonomy development and critical media literacy, particularly during adolescence when parental control strategies become less effective (Valkenburg et al., 1999; Clark, 2011). By encouraging dialogue and perspective-taking, active mediation may foster adolescents’ internalization of behavioral norms and self-regulatory capacities, consistent with self-determination theory (Ryan & Deci, 2017; Padilla-Walker et al., 2012). Among these approaches, growing evidence suggests that active mediation is particularly adaptive in the context of adolescent development.

Recent studies have found that only active mediation helps reduce adolescents’ problematic smartphone use (Chen et al., 2025). However, much of the current literature still relies on questionnaire-based designs to examine how parents’ active mediation relates to adolescents’ online behaviors, often concluding with general recommendations that parents should strengthen active mediation skills. Yet, concrete, practicable strategies or tested training approaches for enhancing parents’ active mediation remain limited in the evidence base (Tan et al., 2024). Specifically, existing programs vary widely in theoretical grounding, with many lacking explicit communication skill components; delivery formats often rely on single-session workshops rather than structured skill-building; outcome measures rarely target active mediation specifically, instead assessing general parenting attitudes or adolescent behavioral changes; and few studies employ controlled designs to establish causal efficacy (Duerager & Livingstone, 2012; Livingstone & Helsper, 2008). Consequently, there remains a significant gap between correlational findings and intervention-based evidence.

In recent years, researchers have increasingly focused on how to help parents engage in effective parenting. Among the various approaches, parent training has been recognized as an evidence-based method for enhancing parental efficacy, improving parenting skills, fostering positive parent–child relationships, and reducing children’s behavioral problems (Brager et al., 2021). Meta-analytic evidence indicates that parent training programs produce moderate to large effects on parenting skills and child outcomes (Menting et al., 2013). However, few studies have examined whether these effects extend to digital parenting contexts, and even fewer have employed experimental designs with active control conditions to establish causal efficacy (Khurana et al., 2015). This limitation is particularly pronounced in research on adolescent media use, where parental mediation interventions remain largely untested.

Among these models, Parent Effectiveness Training (PET), originally developed by Thomas Gordon, is grounded in humanistic psychology and communication theory. PET emphasizes core interpersonal skills such as active listening, I-messages, and collaborative problem-solving, aiming to enhance parental empathy, reduce coercive interaction patterns, and foster mutual respect within the family (Gerber et al., 2016). Although PET was not originally designed for digital media contexts, its theoretical principles align closely with the psychological foundations of active mediation. Specifically, PET may enhance active mediation through several mechanisms: (1) increasing parental self-efficacy in communication; (2) strengthening parents’ responsiveness to adolescents’ emotional and developmental needs; and (3) promoting dialogue-based rather than control-based regulation strategies.

These processes are conceptually consistent with models suggesting that supportive communication fosters adolescents’ internalization of behavioral norms and self-regulatory capacities. Nevertheless, despite the theoretical compatibility between PET principles and active mediation practices, empirical studies directly testing whether structured PET-based training improves parents’ active mediation of smartphone use remain scarce. To address this gap, the present study developed a structured Parent Active Mediation Enhancement Program grounded in the theoretical framework of PET and adapted to the context of adolescent smartphone use. Rather than examining changes in adolescents’ behavior directly, this study focuses on parents’ self-reported active mediation as the primary outcome, thereby targeting a theoretically meaningful and modifiable parenting skill.

Based on this gap, the present study draws upon the framework of parent effectiveness training and prior research findings to design a Parent Active Mediation Enhancement Program. The goal is to explore whether structured parent training can improve parents’ active mediation levels, thereby providing empirical support for future intervention studies.

## 2. Materials and Methods

### 2.1. Participants

A middle school in Hubei Province, China, was selected as the research site. With the school’s approval, the principal investigator contacted the parents of students by telephone to invite them to voluntarily participate in the parent training program. The inclusion criteria were: (a) having a child in grades 7–8 (ages 12–15), (b) the child meeting diagnostic criteria for problematic smartphone use according to the Mobile Phone Addiction Scale (Hong et al., 2012), and (c) no prior participation in parent training programs. The exclusion criteria included parents who were unable to attend weekly sessions due to work or health constraints. A total of 62 parents agreed to participate in the study, including 15 fathers and 47 mothers, with a mean age of 40.47 years (SD = 4.33). The participants were randomly assigned to two groups using a computer-generated randomization sequence with allocation concealment: an experimental group (n = 31; 7 fathers and 24 mothers) and a control group (n = 31; 8 fathers and 23 mothers). None of the participants had previously taken part in any parent training programs.

During the study, two participants in the experimental group withdrew due to work commitments, resulting in a final sample of 29 participants in the experimental group (7 fathers and 22 mothers). No participants withdrew from the control group. An intention-to-treat analysis was not conducted due to the small sample size; however, we compared baseline characteristics between completers and dropouts, finding no significant differences in age, gender, education level, or baseline active mediation scores (all *p*s > 0.05). A 2 × 3 mixed experimental design was adopted in this study, with group (experimental group vs. control group) as the between-subjects variable and test time (pretest, posttest, and follow-up test) as the within-subjects variable. The sample size was estimated using G*Power 3.1.9.7 software. With an effect size of partial η^2^ = 0.25, the calculation indicated that 28 participants would be sufficient to achieve a statistical power of 0.80. Therefore, the final sample of 60 participants met the statistical power requirements for this study.

### 2.2. Experimental Design

A 2 × 3 mixed experimental design was adopted in this study, with group (experimental group vs. control group) as the between-subjects variable and test time (pretest, posttest, and follow-up test) as the within-subjects variable.

The parent training program was delivered by two licensed psychologists (one senior psychologist with 10+ years of experience in family therapy and one doctoral-level psychologist) who had completed a 40 h training workshop on Parent Effectiveness Training (P.E.T.) methodology. Facilitators followed a standardized intervention manual to ensure consistency across sessions. Participants in the experimental group attended a six-week parent training program, held once per week for two hours per session. Each training session took place on Saturday mornings from 9:30 to 11:30 a.m., ensuring that parents’ regular work schedules from Monday to Friday were not affected. After each session, participants received structured homework assignments requiring them to practice specific skills. Homework completion was reviewed at the beginning of each subsequent session. The control group did not receive any intervention and continued with their routine activities. They were offered the opportunity to participate in the training program after the follow-up assessment.

At the end of each training session, participants were asked to evaluate the effectiveness and quality of the course. Upon completion of the six training sessions, both groups completed a posttest assessing parents’ active mediation levels, followed by a follow-up assessment conducted two months later to balance the need to assess maintenance of effects against practical constraints

Based on psychological counseling theory and practical experience, personal change is a gradual process that requires time. Therefore, during the parent training phase, the program focused solely on improving parents’ active mediation abilities and did not require them to implement intervention attempts on their children’s problematic smartphone use.

### 2.3. Instruments

The materials for the parent training sessions were developed based on the Parent Effectiveness Training (P·E·T) manual and its Chinese translation, Parent Effectiveness Training: The Proven Program for Raising Responsible Children (Gordon, 1970). The course strictly followed the educational philosophy of the Parent Effectiveness Training model, and all activities were designed around enhancing parents’ parenting competence. The content and sequence of the six thematic training sessions were organized in accordance with the reference materials of the PET program.

Considering that all participants were likely parents of children with tendencies toward problematic smartphone use, the training curriculum incorporated relevant content on interventions for problematic smartphone use without altering the core structure of the PET model. For example, in the session “Challenges and Opportunities,” the concept of parental mediation was introduced to help parents recognize how their mediation styles might influence their children’s problematic smartphone use. Through group discussions and role-playing exercises, parents were guided to understand the importance of improving active mediation.

In the “Effective Communication” session, discussions on smartphone use were added. Parents were asked to recall real-life scenarios of their children’s smartphone use and were taught to apply three effective communication techniques to discuss and interact with their children regarding smartphone behavior.

The “Children’s Psychological Needs” session focused on psychological factors influencing adolescents’ problematic smartphone use and introduced relevant empirical findings in psychology. Parents were encouraged to pay attention to their children’s basic psychological needs and to help them adjust their cognition toward smartphone use, thereby reducing their tendency toward addiction through need satisfaction.

Finally, in the “Parental Active Mediation” session, smartphone-related topics were incorporated using role-play activities. Parents learned to express their concerns about their children’s problematic smartphone use using the “I-message” technique and were guided to help their children develop rational smartphone use habits.

A detailed outline of the training curriculum is presented in Table 1.

In this study, the Active Mediation Subscale of Smartphone Use was employed to assess participants’ levels of active mediation before and after the training. This subscale was adapted from the Parental Mediation of Internet Use Questionnaire developed by Nikken and Jansz (2014) and revised for the smartphone use context through a rigorous process involving translation, back-translation, and expert review for face and content validity. The subscale consists of 4 items rated on a 5-point Likert scale ranging from 1 (never) to 5 (always). Higher scores indicate greater use of active mediation strategies. The scale demonstrated adequate reliability in prior research with Chinese parents (Cronbach’s α = 0.77). In the present study, Cronbach’s alpha was 0.82 at pretest, 0.85 at posttest, and 0.87 at follow-up. In addition, demographic variables such as age, gender, and income were collected during the pretest. All participants completed three rounds of measurement: before the training (pretest), after the training (posttest), and two months following the completion of the program (follow-up test).

### 2.4. Statistical Analyses

Data were analyzed using SPSS 27.0. First, descriptive analyses were conducted on the pretest, posttest, and follow-up scores of both the experimental and control groups. Then, the pretest scores of the two groups were compared to examine baseline equivalence. Finally, repeated measures analysis of variance (ANOVA) was conducted to assess the immediate and long-term effects of the parent training program.

## 3. Results

### 3.1. Descriptive Analysis

The scores of the experimental group and the control group at the pretest, posttest, and follow-up test are presented in Table 2. For the experimental group, skewness and kurtosis values were −0.42 and −0.18 (pretest), −0.65 and −0.31 (posttest), and −1.25 and 1.89 (follow-up), respectively. The restricted variability at follow-up (SD = 0.21, range = 4.42–5.00) suggests potential ceiling effects. Item-level analysis showed that 86.2% of experimental group participants scored at or above 4.5 on all four items at follow-up, with 62.1% endorsing the maximum score on at least two items.

### 3.2. Test of Pretest Differences Between the Experimental and Control Groups

To examine whether the participants in the experimental and control groups were equivalent at the pretest stage, a multivariate analysis of variance (MANOVA) was conducted with participants’ age, gender, annual family income, educational level, pretest active mediation scores, and problematic smartphone use scores (as reported by adolescents) as dependent variables, and group as the independent variable. The results of the MANOVA indicated that the overall group effect was not significant, *F*_(5, 54)_ = 1.828, *p* = 0.123, partial η^2^ = 0.145.

Further univariate analyses revealed no significant differences between the two groups in terms of age, annual family income, educational level, or pretest active mediation scores (see Table 3). Additionally, a chi-square test was conducted to examine gender distribution, and the results showed no significant difference between the groups, χ^2^(1) = 0.022, *p* = 0.881.

Moreover, measurements of adolescents’ problematic smartphone use in the two groups showed that there was no significant difference between the groups at the pretest stage.

### 3.3. Repeated Measures Analysis of Variance

**Figure 1:**
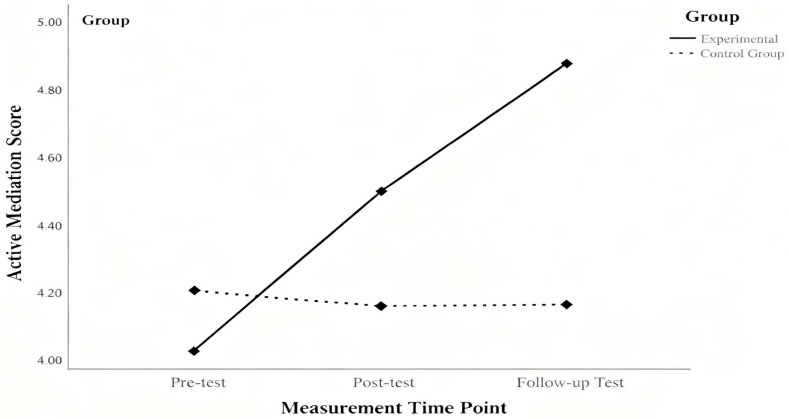
A Graph of the Intervention Effects of Parent Training on Parental Active Mediation.

To examine the effect of parent training on improving parents’ active mediation levels, a 2 (group: experimental vs. control) × 3 (time: pretest, posttest, follow-up) repeated measures analysis of variance (ANOVA) was conducted. Mauchly’s test indicated violation of sphericity, *W* = 0.89, *p* = 0.042; therefore, Greenhouse–Geisser corrections were applied. The results (see Figure 1) showed that the main effect of group was significant, *F*_(1, 58)_ = 4.64, *p* < 0.05, partial η^2^ = 0.074; the main effect of time was also significant, *F*_(2, 57)_ = 22.70, *p* < 0.001, partial η^2^ = 0.44. Moreover, the interaction effect between group and time was significant, *F*_(2, 57)_ = 28.15, *p* < 0.001, partial η^2^ = 0.50.

Simple effects analyses further revealed that there was no significant difference between the experimental and control groups at the pretest stage, *F*_(1, 58)_ = 1.02, *p* > 0.05. However, at the posttest stage, the difference between the training and control groups was significant, *F*_(1, 58)_ = 5.75, *p* < 0.05, partial η^2^ = 0.09. At the follow-up measurement two months later, the difference remained significant, *F*_(1, 58)_ = 40.03, *p* < 0.001, partial η^2^ = 0.41.

These results indicate that parent training can significantly improve parents’ active mediation levels, and the intervention effect remains significant even two months after the training.

## 4. Discussion

Through the pretest–posttest–follow-up design with experimental and control groups, the present study demonstrated that a six-session parent training program can significantly improve parents’ active mediation levels. This finding supports the view that parent training is a cost-effective approach to enhancing parenting skills (Ahadzadeh et al., 2015; Breitenstein & Gross, 2013; Brager et al., 2021). They suggest that interventions targeting adolescents’ problematic smartphone use can consider parent training as an effective approach—first by strengthening parents’ intervention abilities and teaching them active mediation skills and techniques, and then by guiding them to implement these strategies within the family context to reduce their children’s problematic smartphone use.

In family education, one of the most important roles of parents is to intervene in children’s and adolescents’ problematic behaviors (Austin et al., 1999). The effectiveness of such interventions largely depends on parents’ active mediation abilities and levels. Regarding adolescents’ problematic smartphone use, active mediation refers to the practice of engaging in communication and interaction with children about smartphone use in order to reduce their usage (Chen et al., 2025). Previous studies have found that parent–child interaction can influence children’s and adolescents’ cognitive styles (Padilla-Walker et al., 2012), and high-quality parent–child interaction is also a key factor in successfully intervening in youths’ media use (Austin et al., 1999). The PET model, grounded in humanistic psychology, explicitly trains parents in three core skills: active listening, the use of “I-messages,” and the “no-lose” method of conflict resolution (Gordon, 1970). By learning to listen empathetically to their children’s perspectives on smartphone use, express their own concerns without blame or criticism, and collaboratively find mutually acceptable solutions, parents acquire the behavioral repertoire necessary to replace restrictive or monitoring strategies with more constructive dialogues. Thus, the training likely enhanced parental self-efficacy in communication, which in turn facilitated the adoption and maintenance of active mediation behaviors. By effectively improving parents’ active mediation levels through training, this study lays a foundation and provides theoretical support for parental intervention in adolescents’ problematic smartphone use.

Interestingly, active mediation scores in the experimental group continued to rise from posttest to the two-month follow-up, a pattern consistent with a “sleeper effect.” We propose that this delayed enhancement reflects a period of real-world consolidation. The six-week program may have provided the foundational knowledge and initial skill practice. However, the two months following the program likely served as a critical period for parents to experiment with these new skills in authentic, day-to-day interactions with their adolescents. This self-directed application and subsequent reinforcement of successful experiences may have further solidified and even improved their competency, a finding that underscores the importance of post-program practice.

The value of this study lies in its experimental findings, which demonstrate that parent training plays a significant role in enhancing parents’ active mediation levels. First, the results empower parents by showing that their mediation skills are malleable and can be systematically improved through training, increasing their confidence in managing adolescent smartphone use. Second, the supportive group environment of the training allowed parents facing similar challenges to share experiences, reducing feelings of isolation and providing a platform for peer learning. Finally, the sustained effect at follow-up reinforces the need for accessible, long-term support mechanisms, such as online forums or booster sessions, to help parents maintain and continue to develop their skills after the formal training concludes.

### Implications, Limitations, and Future Research

The main limitation of this study is that it focused solely on examining the effect of parent training on parents’ active mediation, assuming that such training could effectively improve parents’ active mediation levels. However, the parent training program used in this study was mainly adapted from the model and content of Parent Effectiveness Training (PET), and no research has yet examined whether this intervention exerts different effects on parental mediation styles and parenting styles. Because parenting style is closely related to parental mediation style—and the latter, as a specific strategy through which parents intervene in children’s and adolescents’ media use, is inevitably influenced by traditional parenting practices (Rosen et al., 2008)—whether parent training has the same impact on these two parenting dimensions was not addressed in this study. Future research could compare interventions targeting parenting style with those focusing on mediation style to further clarify the unique role of mediation-based interventions compared with other parenting programs (Khurana et al., 2015).

Second, there was a gender imbalance among participants, with the majority being mothers. This reflects the reality that mothers assume a larger share of child-rearing responsibilities in most Chinese families. However, parents’ attitudes toward preventing internet addiction may vary depending on both the parent’s and the child’s gender (Liu & Yu, 2013). Future research should aim to recruit more fathers to achieve a balanced gender composition, which would allow for a clearer understanding of how parent training differentially influences fathers’ and mothers’ intervention behaviors.

Third, the evaluation of intervention effects was based on self-reported data from parents. Since participants were highly motivated to change and eager to learn effective strategies to reduce their children’s problematic smartphone use tendencies, the results may have been influenced by social desirability bias. Future studies could adopt more objective evaluation methods, such as observing or videotaping parents’ behaviors during training sessions and having professionals assess improvements in their skills.

Fourth, although each of the experimental and control groups included approximately 30 parents—which is more than the minimum of 14 participants per group required by G*Power analysis—the intervention group consisted of only 29 parents. Moreover, these parents were self-selected volunteers who were strongly motivated to change their children’s problematic behaviors, which could have influenced the intervention outcomes. Therefore, caution should be exercised when generalizing the findings.

Finally, because most parents of adolescents are in middle adulthood, they often lack the time and energy to participate in in-person training programs due to work commitments (Spoth et al., 2000). Additionally, all participants in this study were from urban families. Families in rural areas or those with left-behind children were not included. Parents in such families may be unable to attend in-person sessions due to economic constraints and the necessity of working away from home. Future studies could combine offline and online training formats to make parent training programs more accessible and widely disseminated. 

## 5. Conclusions

(1) Among parents with the same initial level of active mediation, those who participated in the six-session parent training program demonstrated higher levels of active mediation than those who did not.
(2) The active mediation levels of parents who participated in the training remained significantly higher even two months later, indicating that parent training can enhance parents’ active mediation levels over a relatively long term.

## Figures and Tables

**Table 1:** Parent Active Mediation Program Design.

Time	Course Topics	Course Objectives	Course Content	After-Class Exercises
1	Challenges and Opportunities.	1. Understand different types of parental mediation. 2. Increase parents’ awareness of their own intervention patterns. 3. Recognize the importance of adopting active mediation strategies.	1. Group-building and warm-up activities. 2. Challenges parents face when intervening in their children’s smartphone use. 3. Negative effects of problematic smartphone use on adolescents. 4. The role of parental mediation in adolescents’ problematic smartphone use.	Family news sharing.
2	Effective Communication.	1. Enable parents to recognize their daily communication patterns with their children. 2. Understand the negative impact of poor communication on problematic smartphone use. 3. Learn effective parent–child communication techniques.	1. The negative effects of twelve types of poor communication and basic listening skills. 2. Learning parent–child communication techniques. 3. Group practice of communication techniques.	Parents’ self-practice of effective communication techniques at home.
3	Children’s Psychological Needs.	1. Understand children’s psychological needs. 2. Comprehend the relationship between unmet psychological needs and problematic smartphone use. 3. Learn techniques to fulfill children’s psychological needs.	1. Psychological needs and problematic smartphone use. 2. Learning techniques for responding to children’s psychological needs. 3. Group practice of response techniques.	Communicating with children to understand their psychological needs.
4	Parental Active Mediation.	1. Understand the relationship between fear of missing out, positive outcome expectations, and problematic smartphone use. 2. Learn to express feelings and concerns using the “I-message” technique. 3. Learn to apply active mediation strategies to address smartphone use issues.	1. The relationship between fear of missing out, positive outcome expectations, and problematic smartphone use. 2. Group practice using the “I-message” technique 3. Learning active mediation strategies. 4. Resolving conflicts over smartphone use.	Self-practice using the “I-message” technique.
5	Empowered and Positive Family Energy.	Learn to hold family meetings.	Role-playing activities. Simulated family meetings.	Holding family meetings.
6	Wise Parenting Showcase.	Summary Reflection Planning.	1. Work summary. 2. Demonstration of wise parenting skills. 3. Sharing group achievements. 4. Future planning: Family education after the six sessions.	1. Practicing learned communication skills in interactions with children. 2. Reading books related to parent–child education.

**Table 2:** Scores of the Experimental and Control Groups at Pretest, Posttest, and Follow-up (M ± SD).

Group	Time Point	Active Mediation
Experimental Group	Pretest	4.02 ± 0.73
	Posttest	4.50 ± 0.48
	Follow-up Test	4.88 ± 0.21
Control Group	Pretest	4.20 ± 0.64
	Posttest	4.16 ± 0.62
	Follow-up Test	4.16 ± 0.58

**Table 3:** Test of Pretest Differences Between the Experimental and Control Groups.

Variable	Experimental Group (n = 29)	Control Group (n = 31)	*F*_(1, 58)_	Partial η^2^
	*M* ± *SD*	*M* ± *SD*		
Gender (man/female) ^a^	7/22	8/23	0.022	
age	40.38 ± 3.19	40.13 ± 5.09	0.051	0.001
Family Income	2.38 ± 0.73	2.74 ± 0.82	3.287	0.054
Educational Level	2.03 ± 0.57	1.87 ± 0.62	1.136	0.019
Active Mediation (Pretest)	4.02 ± 0.73	4.20 ± 0.64	1.019	0.017
Problematic smartphone use (Pretest)	4.60 ± 0.95	4.40 ± 0.84	0.755	0.013

Note: ^a^ Gender is a dummy variable, with 0 representing male and 1 representing female.

## Data Availability

The authors had full control of all the primary data and the datasets used and analyzed during the current study are available from the corresponding authors on reasonable request.
